# Results from the first Kuwait National Bariatric Surgery Report

**DOI:** 10.1186/s12893-020-00946-x

**Published:** 2020-11-23

**Authors:** Salman Al Sabah, Eliana Al Haddad, Taleb Jumaa, Jasim Al Abbad, Fareed Salam, Mustafa Abbas, Mubarak Al Kandari, Aws Al Ozairi

**Affiliations:** 1 Kuwait University, Kuwait City, Kuwait; 2 Al Amiri Hospital, Kuwait City, Kuwait; 3 Department of Surgery, Faculty of Medicine, Kuwait University, Kuwait City, Kuwait

**Keywords:** Bariatric surgery, National report, Kuwait, Sleeve gastrectomy, Bypass

## Abstract

**Background:**

Currently, more than 30% of the population in the gulf demonstrate a body mass index (BMI) exceeding 30. This burden of obesity has proven to take a toll on the population; therefore, we created the first Kuwait National Bariatric Surgery Database to report on bariatric surgeries performed in Kuwait.

**Methods:**

Data was collected from the six public hospitals in Kuwait. This data was then submitted to a merged National Registry. Data web portal were used to upload, merge, and analyze the data.

**Results:**

The average age for participants was 32.6 years. The average preoperative BMI was 45.9 kg/m^2^ for males and 43.3 kg/m^2^ for females. 16.4% of males and 12.3% of females presented with type 2 diabetes, while the most prevalent obesity related disease was a poor functional status in both males and females (90.8% and 90.5%, respectively). Most procedures performed in Kuwait are sleeve gastrectomy. The most encountered in-hospital complication after primary bariatric surgery was bleeding (1.5%), with Roux-en-Y gastric bypass (RYGB) having the highest recorded rate of post-operative complications (3.6% bleeding). The overall rate of operative complications was 2.6%, which was most prevalent post-RYGB (10.3%) and lowest post-sleeve gastrectomy (2.5%).

**Conclusion:**

The importance of tracking and documenting the journey and change in the rates of obesity and effectiveness of bariatric procedures in individual countries with significantly high obesity rates is imperative to be able to create a plan of action to tackle this worldwide epidemic. This report will be able to provide the population with an accurate accounting that demonstrates further the safety of bariatric/metabolic surgery.

## Background

As of 2006, the number of overweight and obese people had overtaken the number of people with malnutrition in the world [1, 2]. In Europe alone, the prevalence of obesity has shown a threefold increase in the past 2 decades, with obesity currently affecting 150 million adults, 15 million children and causing 1 million deaths annually [3]. This brings into light the role of bariatric surgery in the management of this ever-growing epidemic. Currently, all bariatric procedures have proven to be effective in the treatment of morbid obesity, as well as the comorbidities related to it as compared to its counter non-surgical interventions [4, 5].

When looking at numbers around the world, in 2014, the International Federation for the Surgery of Obesity and Metabolic Surgery of Obesity and Metabolic Disorders (IFSO) was able to show that the number of bariatric and metabolic surgeries performed in the Asia–Pacific Chapter was about a 1/3 of those of the other three chapters [6]. However, this number proved to have increased by 2.5-fold between the years 2011–2014 [6, 7]. This increase was especially noted in the Middle Eastern countries due to the increased burden of obesity, diabetes and metabolic syndrome they are facing there as of recent years [8]. Currently, more than 30% of the population in the gulf region demonstrate a body mass index (BMI) exceeding 30, with Kuwait having 39.7% of its population placed in this obesity range [9]. This burden of obesity has proven to take a toll on the population as a whole, with the 2013 IFSO worldwide Survey showing Kuwait as having the highest number of bariatric surgeries performed as a percentage of the national population, leading with 0.1642%.

Therefore, we thought it was imperative to create the first Kuwait National Bariatric Surgery Database Report to report on bariatric surgeries performed in Government hospitals in Kuwait, baseline obesity-related diseases, operation types, operative outcomes and status after bariatric surgery. It will be the first of its kind in the State of Kuwait, as well as the Gulf and Middle-East, and has the potential to help the bariatric community establish essential benchmark knowledge and outcomes about the patients we are operating upon, their age and gender distributions, body mass index (BMI) and the burden of comorbidities as well as track national trends in surgery over time.

## Methods

### National database data collection

For the report, permission was obtained from the ministry of health to collect data from the six public hospitals in Kuwait on bariatric results and outcomes. Invitations were sent to bariatric surgeons working in these hospitals, of which 63 contributed to the data collection. This data was then submitted to a merged National Registry. A Direct Data Entry system, and an Upload-My-Data web portal were used to upload, merge, and analyze the data. Data was collected on 3302 cases, of which 2704 were primary procedures (Table 1). Data collection was demonstrably of a very high quality; over 87% of entries for patients having their primary operation had either no missing data or one missing data-item amongst a list of 10 obesity-related diseases assessed pre-operatively. All patients provided written consent prior to undergoing their procedure.

**Table 1:** Contributing hospitals

	Count
Hospital	
Al Adan Hospital	290
Al Amiri Hospital	1056
Al Jahra Hospital	410
Al Sabah Hospital	566
Farwaniya Hospital	380
Mubarak Al Kabeer Hospital	600
*All*	*3302*

### Definitions of obesity related diseases

We aimed to set a standard baseline for defining obesity related diseases as to be able to compare between individual patients. Positive responses (data denoting patients who have the condition) were:

1. Type 2 diabetes:
  (i) Impaired glycaemia or impaired glucose tolerance.
  (ii) Insulin treatment.
  (iii) OAD & insulin treatment.
  (iv) Oral hypoglycaemics.
2. Back pain or leg pain:
  (i) Yes
3. Depression:
  (i) Depression on medication.
4. Impaired functional status:
  (i) Can climb 1 flight of stairs without resting.
  (ii) Can climb half a flight of stairs without resting.
  (iii) Walking.
  (iv) Requires wheelchair or is housebound.
5. Gastro-Esophageal Reflux Disease (GERD):
  (i) Daily medication (H2 receptor antagonists (H2RA) or proton pump inhibitors (PPI))
  (ii) Intermittent medication.
  (iii) Intermittent symptoms; no medication.
6. Hypertension:
  (i) Treated hypertension.
  (ii) Untreated hypertension.
7. Dyslipidaemia:
  (i) Dyslipidaemia.
8. Liver disease:
  (i) Fatty liver.
  (ii) Mild steatosis.
  (iii) Severe steatosis.
9. Sleep apnea:
  (i) Yes.
10. Increased risk of deep vein thrombosis (DVT) or pulmonary embolism (PE) contains any one or more of:
  (i) History or risk factor for DVT or PE.
  (ii) Venous edema with ulceration.
  (iii) Vena cava filter.
  (iv) Obesity|hypoventilation syndrome.

### Obesity Surgery Mortality Risk Score

The Obesity Surgery Mortality Risk Score (OSMRS) stratifies patients undergoing bariatric surgery into three categories depending on how many of the following risk factors they possess:

- Male gender.
- Age ≥ 45 years at the time of surgery.
- BMI > 50 kg m^2^.
- Hypertension.
- Risk factors for deep vein thrombosis/pulmonary embolism.

The patient is ascribed one point for each of the above risk factors and a cumulative score determined, giving a total score in the range zero to five; this score is normally grouped into one of three categories:

- Group A: score 0–1 (low risk)
- Group B: score 2–3 (moderate risk)
- Group C: score 4–5 (high risk)

Patients with higher OSMRS, are thought to be at a greater risk of post-operative complications and mortality. The score is only calculated when all of the required data are available in the operation record.

### Data analysis

Descriptive statistics were used for the analysis of the data. The contributors were reassured that no statistical comparison would be attempted between different units. Furthermore, as data from different hospitals may only provide variable representation of the population, no comparative analysis was performed between hospitals.

On the whole, unless otherwise stated, the tables and charts in this report record the number of procedures. The numbers in each table are bolded so that entries with complete data for all of the components under consideration are shown in regular black text. If one or more of the database questions under analysis is blank, the data are reported as unspecified in bold text. The totals for both rows and columns are highlighted as italic text. Some tables record percentage values; in such cases this is made clear by the use of an appropriate title within the table and a % symbol after the numeric value.

Rows and columns within tables have been ordered so that they are either in ascending order or with negative response options first (No; None) followed by positive response options (Yes; One, Two, etc.).

### Graphs

All entries with missing data are excluded from the analysis used to generate the graph. In the charts prepared for this report, most of the bars plotted around rates (percentage values) represent 95% confidence intervals. The width of the confidence interval provides some idea of how certain we can be about the calculated rate of an event or occurrence. If the intervals around two rates do not overlap, then we can say, with the specified level of confidence, that these rates are different; however, if the bars do overlap, we cannot make such an assertion.

Bars around averaged values (such as patients’ age, post-operative length-of-stay, etc.) are classical standard error bars or 95% confidence intervals; they give some idea of the spread of the data around the calculated average. In some analyses that employ these error bars there may be insufficient data to legitimately calculate the standard error around the average for each sub-group under analysis; rather than entirely exclude these low-volume sub- groups from the chart their arithmetic average would be plotted without error bars. Such averages without error bars are valid in the sense that they truly represent the data submitted; however, they should not to be taken as definitive and therefore it is recommended that such values are viewed with extra caution.

## Results

### Patient demographics

**Fig. 1:**
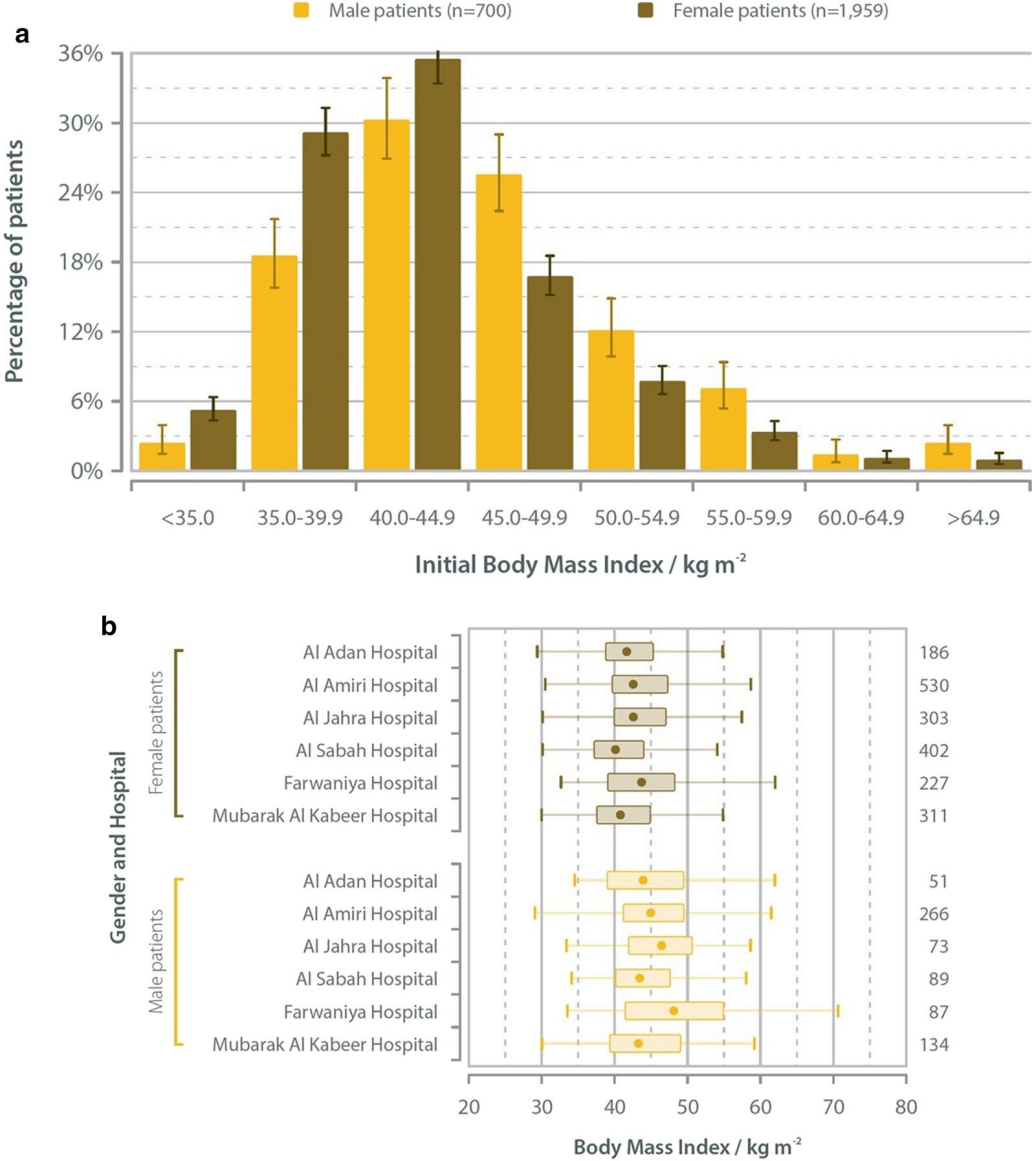
**a** Primary surgery: initial BMI and gender. **b** Primary surgery: initial BMI and gender at each hospital

73.6% of all patients were seen to be female. The average age for participants was 32.6 years, with the average age for male patients being 32.6 years; and for female patients 32.6 years. The majority of patients were under the age of 35 years and this can be accounted for by the fact that Kuwait has a generally younger population compared to the rest of world. In addition, the national policy stipulates that patients over 65 may not be offered this kind of intervention (Table 2). The average preoperative BMI was 45.9 kg/m^2^ for male patients and 43.3 kg/m^2^ for female patients (Fig. 1a). The distribution of BMI was further sub-divided by hospital, which can be seen in Fig. 1b.

**Table 2:** Primary surgery: age and gender

	Gender			
	Male	Female	Unspecified	All
Age/years				
< 16	4	20	**0**	24
16–20	106	286	**3**	395
21–25	111	334	**2**	447
26–30	99	283	**2**	384
31–35	123	316	**4**	443
36–40	90	253	**3**	346
41–45	66	182	**2**	250
46–50	59	151	**2**	212
51–55	24	82	**1**	107
> 55	22	62	**1**	85
Unspecified	**4**	**7**	**0**	**11**
*All*	*708*	*1976*	***20***	*2704*

### Obesity related diseases

**Fig. 2:**
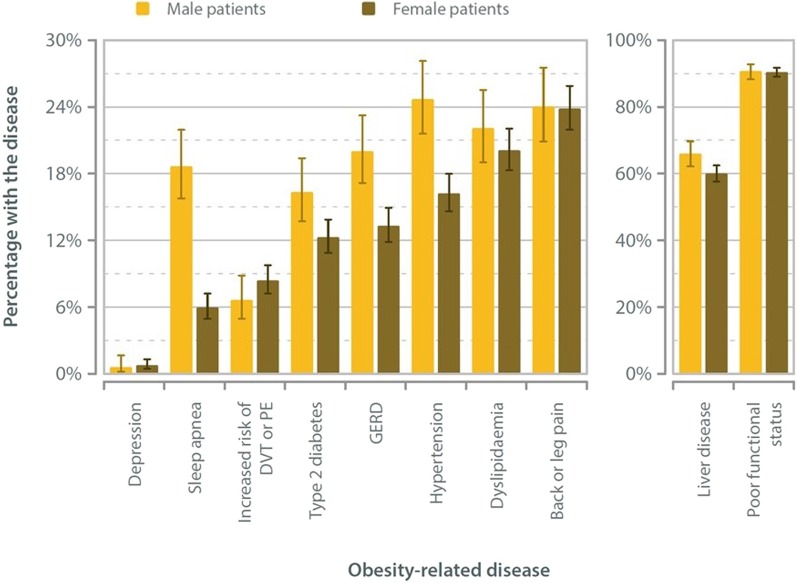
Primary surgery: obesity-related disease rates

The Kuwait National Bariatric Surgery Registry records the status of 10 obesity-related diseases for each patient. These 10 obesity-related diseases are: Type 2 diabetes; Back pain or leg pain; Depression; Impaired functional status; Gastro-esophageal reflux disorder (GERD); Hypertension; Dyslipidemia; Liver disease; Sleep apnea; Increased risk of deep vein thrombosis (DVT) or pulmonary embolus (PE) (Table 3, Fig. 2).

**Table 3:** Primary surgery: obesity-related disease and gender

	Male			Female rates				
	No	Yes	Unspecified	No	Yes	Unspecified	Male	Female
Back or leg pain	505	160	**43**	1413	443	**120**	24.1%	23.9%
Depression	663	4	**41**	1918	15	**43**	0.6%	0.8%
Type 2 diabetes	573	112	**23**	1677	235	**64**	16.4%	12.3%
Increased risk of DVT or PE	646	46	**16**	1777	163	**36**	6.6%	8.4%
Poor functional status	63	621	**24**	180	1709	**87**	90.8%	90.5%
GERD	559	140	**9**	1699	261	**16**	20.0%	13.3%
Hypertension	524	172	**12**	1615	313	**48**	24.7%	16.2%
Dyslipidaemia	511	145	**52**	1450	365	**161**	22.1%	20.1%
Liver disease	213	414	**81**	661	995	**320**	66.0%	60.1%
Sleep apnea	523	120	**65**	1667	106	**203**	18.7%	6.0%

**Fig. 3:**
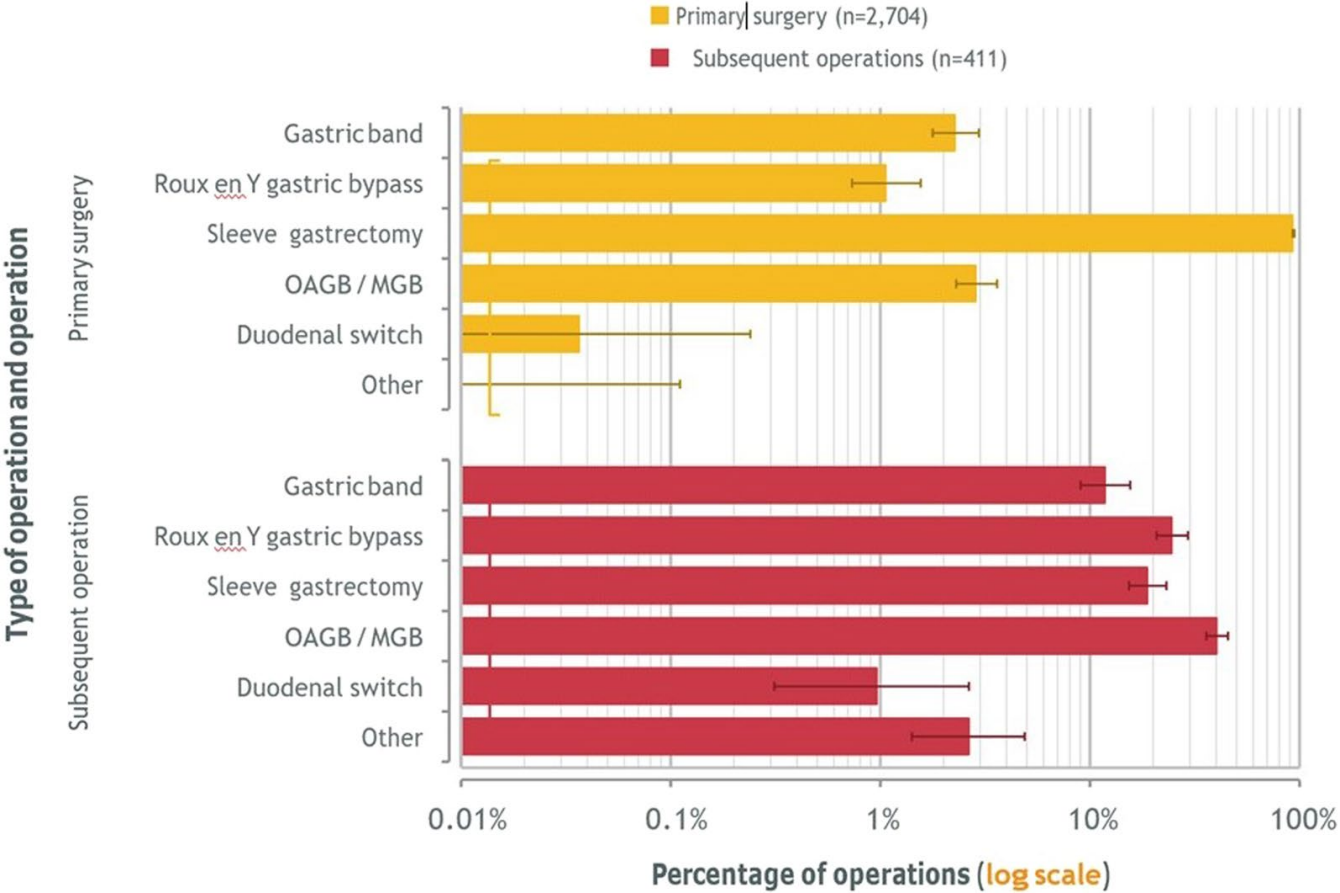
Type of operation and operation performed

As shown in the Table 4 and Fig. 3, male bariatric surgery patients in Kuwait tend to have higher rates of obesity-related disease. 16.4% of males and 12.3% of females presented with type 2 diabetes, while the most prevalent obesity related disease was seen to be a poor functional status in both males and females (90.8% and 90.5%, respectively). Interestingly, rates of medication for depression are very low in absolute terms.

**Table 4:** Primary surgery: Obesity Surgery Mortality Risk Score (OSMRS)

	Analysis			
	Gender			
	Male	Female	Unspecified	All
Obesity Surgery Mortality Risk Score				
0	0	1150	**0**	1150
1	340	485	**0**	825
2	217	198	**0**	415
3	96	36	**0**	132
4	17	8	**0**	25
5	3	0	**0**	3
Group A (0–1)	340	1635	**0**	1975
Group B (2–3)	313	234	**0**	547
Group C (4–5)	20	8	**0**	28
Unspecified	**35**	**99**	**20**	**154**
*All*	*708*	*1976*	***20***	*2704*

### Obesity Surgery Mortality Risk Score

Table 4 demonstrates the OSMRS for our population in Kuwait. The average OSMRS is obviously higher for the male patients (1.70 versus 0.54 for the female patients) as male gender is a component of the scoring system itself. However, very few patients fall in group C (3.0% of male patients and 0.4% of female patients).

### Surgery

The vast majority of procedures performed in Kuwait are sleeve gastrectomy, which reflects current global trends; single anastomosis gastric bypass (OAGB/MGB) is the second most common procedure. When looking at revisional surgeries, a single anastomosis gastric bypass has mainly been performed as a subsequent procedure, most commonly after a sleeve gastrectomy (Fig. 3, Table 5).

**Table 5:** Types of previous operations to revision surgery

Previous operation	Current (revision) operation	Count
Gastric band	Gastric band	48
	Roux en Y gastric bypass	5
	Sleeve gastrectomy	43
	OAGB/MGB	8
	Other	1
Roux en Y gastric bypass	Roux en Y gastric bypass	8
	Other	2
Sleeve gastrectomy	Gastric band	1
	Roux en Y gastric bypass	79
	Sleeve gastrectomy	30
	OAGB/MGB	144
	Duodenal switch	4
	Other	5
OAGB/MGB	Roux en Y gastric bypass	5
	Sleeve gastrectomy	1
	OAGB/MGB	5
Gastric plication	Roux en Y gastric bypass	2
	OAGB/MGB	4
Others	Roux en Y gastric bypass	1
	Sleeve gastrectomy	1
	OAGB/MGB	5
	Other	2
**Unspecified prior operation**	Roux en Y gastric bypass	2
	Sleeve gastrectomy	3
	OAGB/MGB	1
	Other	1
*All*		*411*

The majority of these procedures are carried out laparoscopically, constituting 94% of the procedures. Open procedures are very rare (0.1%) and this approach is generally only employed in a small minority of revisional procedures (Table 6). This is all in line with current practice across the world. Staple line reinforcements were used in 55.6% of the cases, with the majority being TRS reinforcements (26.2%). Moreover, the majority of surgeons (83.6%) used a 36 Fr bougie for their operations.

**Table 6:** Type of operation and operative approach

	Type of operation				
	Balloon	Primary	Revision	Unspecified	All
Operative approach					
Counts					
Laparoscopic	19	2687	402	**0**	*3108*
Laparoscopic converted to open	0	1	2	**0**	*3*
Endoscopic	167	3	1	**0**	*171*
Open	0	0	3	**0**	*3*
Unspecified	**1**	**13**	**3**	**0**	***17***
All	*187*	*2704*	*411*	***0***	*3302*
Percentages					
Laparoscopic	10.2%	99.9%	98.5%		*94.6%*
Laparoscopic converted to open	0.0%	0.0%	0.5%		*0.1%*
Endoscopic	89.8%	0.1%	0.2%		*5.2%*
Open	0.0%	0.0%	0.7%		*5.2%*

Gallbladder and hernia repair were the most common additional procedures performed at the time of bariatric procedures (Table 7).

**Table 7:** Additional procedures performed alongside the primary surgery

	Additional procedures				Details of additional procedures			
	No	Yes	Unspecified	All	Cholecystecomy	Hernia repair	Liver biopsy	Other
Gastric band	59	1	**2**	*62*	0	0	0	1
Roux en Y gastric bypass	25	4	**0**	*29*	0	4	0	0
Sleeve gastrectomy	2324	160	**50**	*2534*	73	70	3	23
OAGB/MGB	70	6	**2**	*78*	4	1	0	1
Duodenal switch	1	0	**0**	*1*	0	0	0	0
All	*2479*	*171*	***54***	*2704*	*77*	*75*	*3*	*25*
Gastric band		1.7%			0.0%	0.0%	0.0%	1.7%
Roux en Y gastric bypass		13.8%			0.0%	13.8%	0.0%	0.0%
Sleeve gastrectomy		6.4%			2.9%	2.8%	0.1%	0.9%
OAGB/MGB		7.9%			5.3%	1.3%	0.0%	1.3%
Duodenal switch		0.0%			0.0%	0.0%	0.0%	0.0%
*All*		*6.5%*			*2.9%*	*2.8%*	*0.1%*	*0.9%*

### Operative complications

The most encountered in-hospital complication after primary bariatric surgery was seen to be bleeding (1.5%). When it came to looking at post-operative complications, Roux-en-Y gastric bypass has the highest recorded rate of post-operative complications, with 3.6% of patients presenting with bleeding.

On average most patients were discharged home within 2–3 days after their bariatric procedure.

**Fig. 4:**
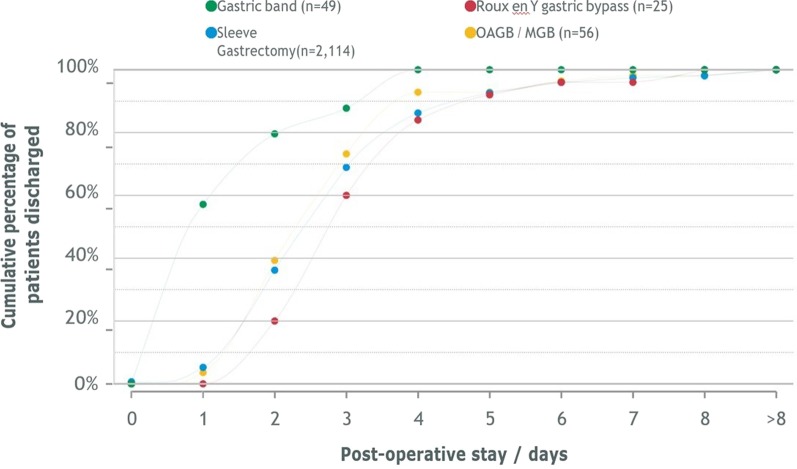
Primary surgery: post-operative stay and operation

Patients tend to go home sooner after a gastric band procedure (over 50% discharged by post-operative day 1) than after undergoing other procedures (Fig. 4).

The overall rate of operative complications was seen to be 2.6%. The highest rate of operative complications was after Roux en Y gastric bypass (10.3%; 29 patients) and the lowest after sleeve gastrectomy (2.5%; 61 patients). (Table 8).

**Table 8:** Complications encountered following primary surgery

	Complications recorded			Complications details				
	No	Yes	Unspecified	Leak	Bleed	Staple line leak	Other	Unspecified
Gastric band	60	2	**1**	0	0	0	1	**0**
Roux en Y gastric bypass	27	1	**0**	0	1	0	0	**1**
Sleeve gastrectomy	2476	57	**47**	0	38	3	12	**1**
OAGB/MGB	76	1	**1**	0	1	0	0	**1**
Duodenal switch	1	0	**0**	0	0	0	0	**0**
All	*2640*	*61*	***49***	*0*	*40*	*3*	*13*	***3***

	Leak	Bleed	Staple line leak	Other	Unspecified
Gastric band	0.0%	0.0%	0.0%	1.6%	**0.0%**
Roux en Y gastric bypass	0.0%	3.6%	0.0%	0.0%	**3.6%**
Sleeve gastrectomy	0.0%	1.5%	0.1%	0.5%	**0.0%**
OAGB/MGB	0.0%	1.3%	0.0%	0.0%	**1.3%**
Duodenal switch	0.0%	0.0%	0.0%	0.0%	**0.0%**
All	*0.0%*	*1.5%*	*0.1%*	*0.5%*	***0.1%***

Furthermore, this report demonstrates that bariatric procedures in Kuwait are safe for patients with a 0.0% reported in-hospital operative mortality rate. The major cardiac complication encountered post-operatively was Dysrhythmia, encountered in 15.5% of patients, with the majority (9.5%) seen in post Roux-en-Y patients. The only other major complication encountered was vomiting/poor intake post-operatively, seen in 18.9% of patients. This can be seen in Table 9.

**Table 9:** Operative complications recorded post-bariatric surgery

	No	Yes	Unspecified	Complication rate
Complication recorded				
Operative complications				
Gastric band	59	2	**1**	3.3% (0.6–12.4%)
Roux en Y gastric bypass	26	3	**0**	10.3% (2.7–28.5%)
Sleeve gastrectomy	2426	61	**47**	2.5% (1.9–3.2%)
OAGB/MGB	75	2	**1**	2.6% (0.5–9.9%)
Duodenal switch	1	0	**0**	0.0% (0.0–95.0%)
All	*2587*	*68*	***49***	*2.6% (2.0–3.3%)*
Cardio-vascular complications				
Gastric band	40	1	**21**	2.4% (0.1–14.4%)
Roux en Y gastric bypass	18	3	**8**	14.3% (3.8–37.4%)
Sleeve gastrectomy	1857	97	**580**	5.0% (4.1–6.0%)
Duodenal switch	1	0	**0**	0.0% (0.0–95.0%)
All	*1967*	*105*	***632***	*5.1% (4.2–6.1%)*
Other complications				
Gastric band	39	3	**20**	7.1% (1.9–20.6%)
Roux en Y gastric bypass	19	3	**7**	13.6% (3.6–36.0%)
Sleeve gastrectomy	1815	141	**578**	7.2% (6.1–8.5%)
OAGB/MGB	49	6	**23**	10.9% (4.5–22.9%)
Duodenal switch	1	0	**0**	0.0% (0.0–95.0%)
All	*1923*	*153*	***628***	*7.4% (6.3–8.6%)*
In-hospital mortality				
Operation				
Gastric band	62	0	**0**	0.0% (0.0–4.7%)
Roux en Y	29	0	**0**	0.0% (0.0–9.8%)
Sleeve gastrectomy	2534	0	**0**	0.0% (0.0–0.1%)
OAGB/MGB	78	0	**0**	0.0% (0.0–3.8%)
Duodenal switch	1	0	**0**	0.0% (0.0–95.0%)
All	*2704*	*0*	***0***	*0.0% (0.0–0.1%)*

## Discussion

Achieving a milestone not to be celebrated, Kuwait finds itself as having one of the highest prevalence of obesity in the world [9]. In addition, Kuwait ranks first for the number of bariatric / metabolic procedures performed as a percentage of her population. In essence, the disease and the treatment could overwhelm the ability and resources to control it. Therefore, to maintain control of the situation, a wide range of data must be collected, stored in a national database, and analyzed. The Kuwaiti First National Bariatric Surgery Database Report was prepared to provide a full accounting of the practice of bariatric/metabolic surgery throughout Kuwait. This report captures a wide range of data from all of the government hospitals where bariatric and metabolic surgery is being performed. Therefore, this collected and configured data will be invaluable for understanding the current status and trends and enable the leaders to prepare best for the future.

In the general population in Kuwait there are approximately as many obese men as there are obese women. Despite this, as previously demonstrated, about three-quarters of the surgical patient population are female. A recent analysis (2018) from the International Federation for the Surgery of Obesity and Metabolic Disorders (IFSO) Global Registry [10] shows that Kuwait’s experience is in line with what is reported in other countries; in fact, Kuwait’s percentage of female patients falls right in the middle of the global rankings. Furthermore, this recent report was able to further demonstrate that Kuwait has one of the youngest patient populations in the world, while Germany demonstrated one of the oldest. When looking at pre-operative weight and BMI, the IFSO global registry report was able to show that the patient population in Kuwait undergoing primary surgery falls right in the middle of the ordered distribution of BMIs by country, with Germany once again occupying the higher end.

The reported rates of obesity-related disease vary from region to region, and from country to country. The data we collected puts the reported rates in the Middle East in a wider context, and allows us to compare the rates we see in Kuwait to other regions. Generally, rates of most associated obesity-related conditions that we investigated are low in the Middle East compared to other regions; in the Kuwaiti population some rates are relatively low compared to the average for the Middle East as a whole (hypertension, depression, sleep apnea) [10]. Furthermore, the Kuwaiti bariatric surgery patient population falls at the low-risk end of the spectrum, according to OSMRS grouping, when compared to the other countries in the IFSO Global Registry, with close to 30% of patients from Georgia occupying the Group C denomination compared to the 1% from the Kuwaiti population.

When it came to looking at the bariatric surgeries performed, data from the IFSO Global Registry demonstrate that sleeve gastrectomy is the most commonly performed bariatric surgery operation in many countries. Where sleeve gastrectomy is less common, patients tend to have a Roux-en-Y gastric bypass operation instead. The data from Kuwait fall in the portion of the ordered distribution where rates of sleeve gastrectomy are very high. Australia, however, reported that 100% of the surgeries performed were LSG, while in Lithuania, the majority of surgeries performed were a Roux-en-Y operation, and OAGB/MGB dominated in Kazakhstan [10].

Comparing post-operative length-of-stay from the IFSO Global Registry and the current data from the Kuwait National Bariatric Surgery Registry was able to demonstrate that after sleeve gastrectomy, half of patients in the IFSO Registry are discharged by around 1 day after the operation, whereas half of bariatric surgery patients in Kuwait are discharged by about 2 days after surgery. However, within 5 days of treatment, over 90% of patients have been discharged The differences in the two patterns of post-operative stay for the patients who have had an OAGB/MGB are quite small, especially when one considers the fact that the number of Kuwaiti patients treated using this technique is quite low at the moment, which means that the observed distribution will almost certainly change as more data are accumulated for this group.

## Limitations

Limitations of the paper include lack of generalizability due to incomplete case ascertainment, and inability to assess incomplete or erroneous data submission, as well as incomplete follow-up data. In addition to the limitations of the Yes/No questions, there is unknown selection bias for those patients with recorded follow-up. Long-term survival is currently not known and there is a desire to collect long-term follow-up data for all patients operated upon in Kuwait. In addition some patients are transferred from other hospitals for management of complications of surgery carried out elsewhere. The outcomes of these patients are not currently collected nor are they reported here in this Report, but it would be desirable to track the outcomes of these patients in the future. Nevertheless, it is very gratifying to see that there were no reported in-hospital deaths for any patients undergoing bariatric surgery at Government hospitals during the period of study of the Registry.

## Conclusion

The burden of obesity-related disease in the operated populations is high but varies greatly between regions. The importance of tracking and documenting the journey and change in the rates of obesity and effectiveness of bariatric procedures in individual countries with significantly high obesity rates is imperative to be able to create a plan of action to tackle this worldwide epidemic. With Kuwait being one of the countries with the highest rate of obesity and bariatric surgeries, the challenges faced in the creation of this report are negligible compared to the benefits it will provide for the future of its population. Furthermore, this report will be able to provide the population an accurate accounting that would be able to demonstrate further the safety of bariatric/metabolic surgery on a generalizable basis.

## Data Availability

The datasets used and/or analyzed during the current study are available from the corresponding author on reasonable request.

## Abbreviations

BMI: Body mass index
RYGB: Roux-en-Y gastric bypass
IFSO: International Federation for the Surgery of Obesity and Metabolic Surgery of Obesity and Metabolic Disorders
H2RA: H2 receptor antagonists
PPI: Proton pump inhibitors
DVT: Deep vein thrombosis
PE: Pulmonary embolus
OSMRS: Obesity Surgery Mortality Risk Score
GERD: Gastro-esophageal reflux disorder
OAGB/MGB: Single anastomosis gastric bypass
